# A Bayesian experimental autonomous researcher for mechanical design

**DOI:** 10.1126/sciadv.aaz1708

**Published:** 2020-04-10

**Authors:** Aldair E. Gongora, Bowen Xu, Wyatt Perry, Chika Okoye, Patrick Riley, Kristofer G. Reyes, Elise F. Morgan, Keith A. Brown

**Affiliations:** 1 Department of Mechanical Engineering, Boston University, Boston, MA 02215, USA.; 2 Google Research, Mountain View, CA 94043, USA.; 3 Department of Materials Design and Innovation, University at Buffalo, Buffalo, NY 14260, USA.; 4 Department of Biomedical Engineering, Boston University, Boston, MA 02215, USA.; 5 Division of Materials Science and Engineering, Boston University, Boston, MA 02215, USA.; 6 Physics Department, Boston University, Boston, MA 02215, USA.

## Abstract

While additive manufacturing (AM) has facilitated the production of complex structures, it has also highlighted the immense challenge inherent in identifying the optimum AM structure for a given application. Numerical methods are important tools for optimization, but experiment remains the gold standard for studying nonlinear, but critical, mechanical properties such as toughness. To address the vastness of AM design space and the need for experiment, we develop a Bayesian experimental autonomous researcher (BEAR) that combines Bayesian optimization and high-throughput automated experimentation. In addition to rapidly performing experiments, the BEAR leverages iterative experimentation by selecting experiments based on all available results. Using the BEAR, we explore the toughness of a parametric family of structures and observe an almost 60-fold reduction in the number of experiments needed to identify high-performing structures relative to a grid-based search. These results show the value of machine learning in experimental fields where data are sparse.

## Teaser

Automated testing, Bayesian optimization, and additive manufacturing combine for the autonomous design of structures.

## INTRODUCTION

The processes by which mechanical structures are designed have evolved to include a variety of computational tools that have been successful in producing structures with highly tuned properties (*1*–*10*). However, realizing high-performance mechanical structures often involves optimizing properties that cannot be reliably and rapidly predicted using computation, namely, nonlinear mechanical properties (*11*–*16*). Phenomena such as dynamic self-contacts during large deformation and the dominance of stochastic defects in determining failure in real samples make computation difficult and necessitate experiments. Additive manufacturing (AM) has compounded this problem by both vastly increasing the available design space and introducing a host of previously unknown defects for which researchers and practitioners do not have the benefit of empirical engineering guidelines built on decades of intense study (*17*–*19*). This raises the question of how best to design and optimize structures for properties that are difficult to simulate. One approach that has been successful in chemistry, biology, and, more recently, materials science has been autonomous research in which experiments are selected by machine learning and carried out without human intervention (*20*–*24*). Autonomous research systems have been beneficial in these fields because many properties of interest must be experimentally determined, the vast size of the parameter space limits the effectiveness of brute-force experimentation, and the necessary experiments are compatible with automation. However, autonomous research systems are highly specific to certain classes of experiments and have not been realized in the mechanical domain. Moreover, most prior experimental autonomous research systems have not used Bayesian optimization (BO) to guide the selection of experiments, although simulations have revealed that using BO would be more efficient (*25*–*28*).

**Fig. 1:**
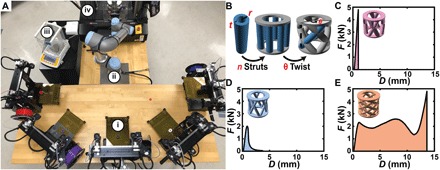
BEAR for studying the mechanics of additively manufactured components. (**A**) Experimental system composed of (i) five dual extruder fused deposition modeling (FDM) printers (M3, MakerGear), (ii) a six-axis robotic arm (UR5e, Universal Robotics), (iii) a scale (CP225D, Sartorius), and (iv) a universal testing machine (5965, Instron Inc.). (Photo credit: Aldair E. Gongora and Bowen Xu, Boston University). (**B**) Model “crossed barrel” family of parametric structures with two circular platforms that are held apart by a series of *n* hollow columns of outer radius *r* and thickness *t* and that are twisted with an angle θ. Force *F* and corresponding displacement *D* from the testing of (**C**) a crossed barrel that did not yield before ~5 kN (designated too strong), (**D**) a crossed barrel that failed in a brittle manner (designated “brittle”), and (**E**) a crossed barrel that exhibited appreciable strength after an initial yield point (designated “ductile”).

Here, we test the hypothesis that combining automated experimentation and BO can accelerate the pace of structural design. Conceptually, realizing a Bayesian experimental autonomous researcher (BEAR) involves two steps: the development of an automated system that performs experiments without human intervention and the incorporation of active learning to choose subsequent experiments in a Bayesian framework. First, we report the design and realization of a mechanical testing system that automatically three-dimensionally (3D) prints and tests parts to determine their mechanical properties such as toughness (Fig. 1A). The high-throughput nature of this system, relative to manual testing, allows for the comprehensive exploration of a large family of structures (Fig. 1B) and the determination of uncertainty inherent to AM using thousands of experiments, a previously impractical concept. Using this experimental data, we run a series of simulations to find that BO should use experiments more efficiently than grid-based searching. Subsequently, we instruct the BEAR to perform experimental campaigns and find that these campaigns resulted in higher-performance structures than those identified through a grid-based campaign that involved 18 times more experiments than were allotted to the BEAR. Last, rather than evaluating a campaign by the required number of experiments, we investigate how campaign duration can be reduced by multiple printers acting in parallel in a multiagent approach and find that the BEAR identifies high-performing structures within 24 hours. Collectively, this work shows the potential for BEARs to affect fields where computational tools are imperfect and experiments are slow and complex.

## RESULTS

### Automating mechanical testing of additively manufactured parts

Toughness is difficult to optimize because it requires maximizing a combination of two properties that tend to be inversely correlated, namely, strength and ductility (*14*, *15*). Defined as the area under the force (*F*)–displacement (*D*) curve, toughness represents how much energy a component can absorb before failure, which makes it an important property to optimize in the context of design for safety and failure tolerance (*29*, *30*). Further compounding the design challenge, it is commonly important to maximize toughness while not exceeding a specified force threshold to avoid damaging more sensitive elements elsewhere in the system. For example, the crumple zone of a car is designed to maximize toughness by absorbing the impact of a collision while not transmitting harmful reactionary forces to the passengers. Because of the importance of dynamic self-contacts, the stochastic influence of defects, and other variations from processing, computational optimization of toughness is extremely difficult (*13*, *16*). To illustrate this, we designed a crossed barrel family of structures (Fig. 1B) with two platforms that are held apart by *n* hollow columns of outer radius *r* and thickness *t* and that are twisted with an angle θ. Structures in this family include those with a wide array of *F*-*D* responses, including structures that exceed a ~5-kN force threshold before yielding (Fig. 1C) and weak structures that fail in a brittle manner (Fig. 1D). The inclusion of a force threshold, as well as the subsequent definition of structure as “too strong,” was incorporated to reflect the presence of a force constraint in designing for toughness, such as in the design of crumple zones. Considering that superlative toughness requires both high ductility and high strength, the best crossed barrel in terms of toughness is not simple to predict. Crossed barrels with high toughness exhibit complex *F*-*D* responses (Fig. 1E) with a number of reentrant contacts and local buckling events.

Acknowledging that toughness needs to be evaluated using experiment, we sought to explore the degree to which the pace of mechanical testing could be accelerated. In particular, we designed and constructed an automated testing system that combines AM, robotics, and mechanical testing (movie S1). In particular, fused deposition modeling (FDM) 3D printers are among the most commonly used 3D printers due to their low cost, versatility, and reliability. Furthermore, FDM-printed parts can be used without additional processing, enabling rapid testing. Thus, five dual extruder FDM printers (M3, MakerGear) were positioned in the working radius of a six-axis robotic arm (UR5e, Universal Robotics). To perform testing and characterization of parts, a scale (CP225D, Sartorius) and a universal testing machine (5965, Instron Inc.) were also positioned in the working radius of the arm. All instruments were coordinated using custom software (MATLAB) (fig. S1).

**Fig. 2:**
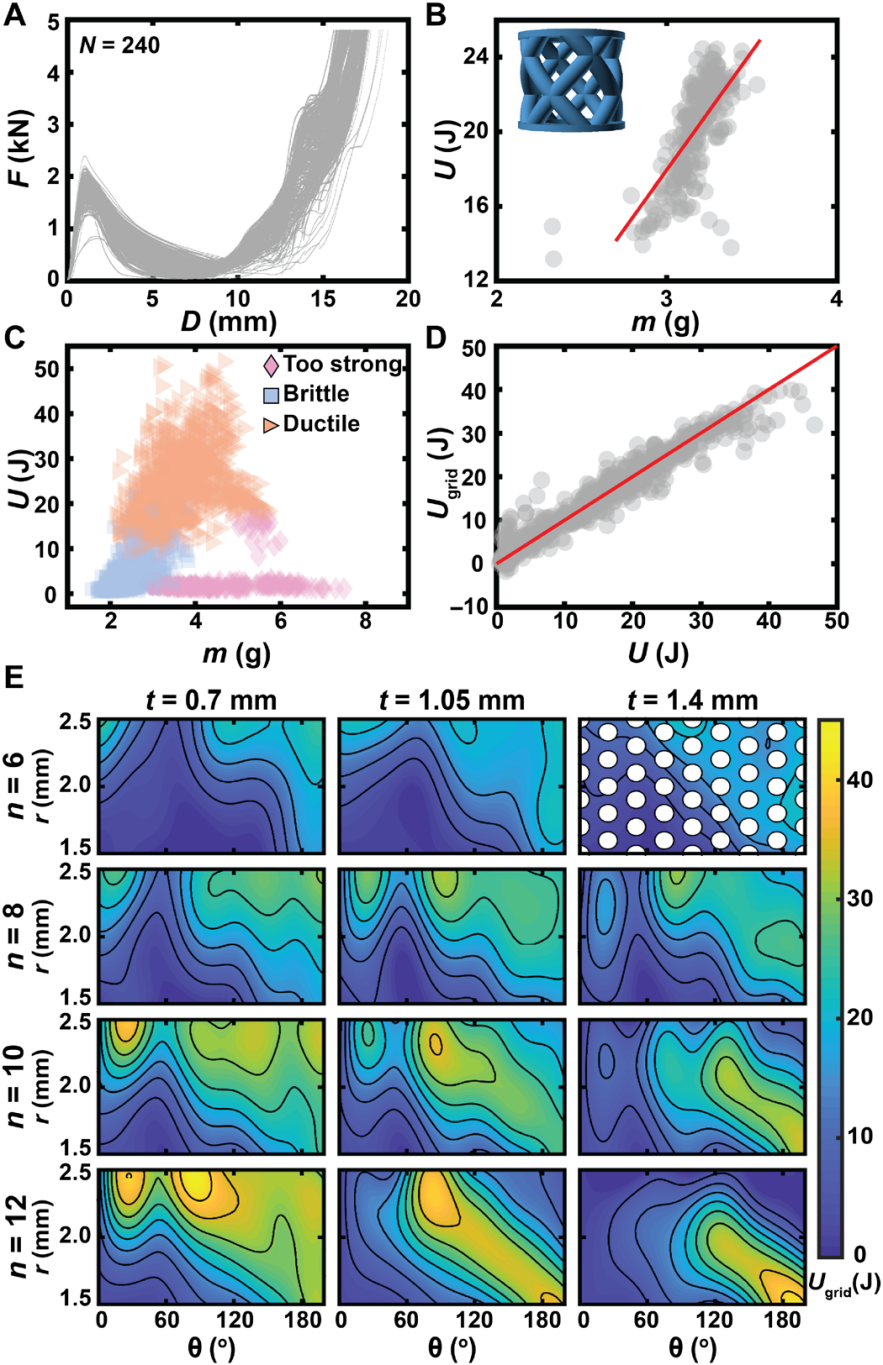
Experimental exploration of the toughness of a family of parametric structures. (**A**) Overlaid *F* versus *D* curves for 240 samples printed with *x* = (*n*, θ, *r*, *t*) = (8,100 ° ,2 mm,1.05 mm). (**B**) Experimental toughness *U* versus component mass *m* for the samples shown in (A). Red line denotes a linear fit with a correlation coefficient of 0.71. (**C**) *U* versus *m* for 1800 samples taken in a grid across the entire parameter space. Marker shape denotes the category of mechanical response. (**D**) Predicted toughness *U*_grid_ based on a Gaussian process regression (GPR) trained on the 1800 experimental data points evaluated at *x* versus average *U*(*x*). The red line has zero intercept and a slope of one as a guide to the eye. (**E**) Surface plot of *U*_grid_ across the entire 4D parameter space with the discretization of the experimental grid represented as white circles in the top right panel.

Before undertaking more complex design or optimization processes, it is necessary to consider that quality control is a pervasive challenge in AM. The extensive exploration of manufacturing uncertainty can be onerous in some cases; however, the automated testing system provided an avenue for rapidly quantifying the uncertainty inherent to properties such as toughness. Thus, we initially performed a series of experiments in which the same design was printed and tested 240 times using all available printers (Fig. 2A) and the mass of each part— measured in situ—had a standard deviation (SD) equal to 4.6% of the mean. The toughness *U* was found to have an SD equal to 12.8% of the mean (Fig. 2B). Because of the empirical nature of these quantities, it would be difficult or impossible to predict the sensitivity with which toughness depends on mass. Having this vast dataset, which is made possible by the high-throughput nature of the system, allows us to approximate the variation in toughness that is uncorrelated with mass, which we find to be 5.8% of the mean. The individual printer mass and toughness variations are reported in fig. S2. This study allowed for the exploration of the correlation between these two properties (Fig. 2B), revealing a correlation coefficient of 0.71 between *U* and *m*, indicating that measurable deviations in print outcome are at least partially responsible for the observed variation in mechanical behavior. The fit in Fig. 2B is insensitive to the removal of the seemingly spurious data.

### High-throughput experimentation and sequential design selection

Despite the large observed variability in performance, it is conceivable that the high-throughput nature of this research platform could allow for sufficient experiments to empirically identify an optimal design within a family of structures. To test this, we performed a grid-based experimental campaign in which 600 distinct designs were tested in triplicate without human intervention (fig. S3). During this automated experimental campaign, a wide variation in *U* was observed, ranging from 0.3 to 51.5 J with a mean value of 15.3 J (Fig. 2C). To estimate the value and uncertainty of the experimental response *U*(*x*), where *x* = (*n*, θ, *r*, *t*) was approximated using a Gaussian process regression (GPR) model *U*_grid_ with a squared exponential kernel (Fig. 2D) (*25*). *U*_grid_ revealed a complex response with several high-performing regions (Fig. 2E) and a predicted optimum of 43.4 ± 6.0 J at (12,85°, 2.45 mm, 0.7 mm).

While brute-force experimentation allowed us to predict an optimum design, the active learning community has shown through simulations that sequentially selecting experiments using BO finds optima using fewer samples. The BO framework is composed of two components, a belief model that captures the relationship between parameters and response and a decision-making policy that guides the selection of experiments (*25*). While *U*(*x*) is ultimately an experimentally observed function, *U*_grid_(*x*) represents an approximation of *U*(*x*) that can be used to evaluate BO strategies in simulation. Thus, we performed a series of simulations using the BO framework with *U*_grid_(*x*) treated as a surrogate for the ground truth *U*(*x*). To approximate experimental variations, we added a zero-mean Gaussian noise with SD σ to each simulated measurement.

**Fig. 3:**
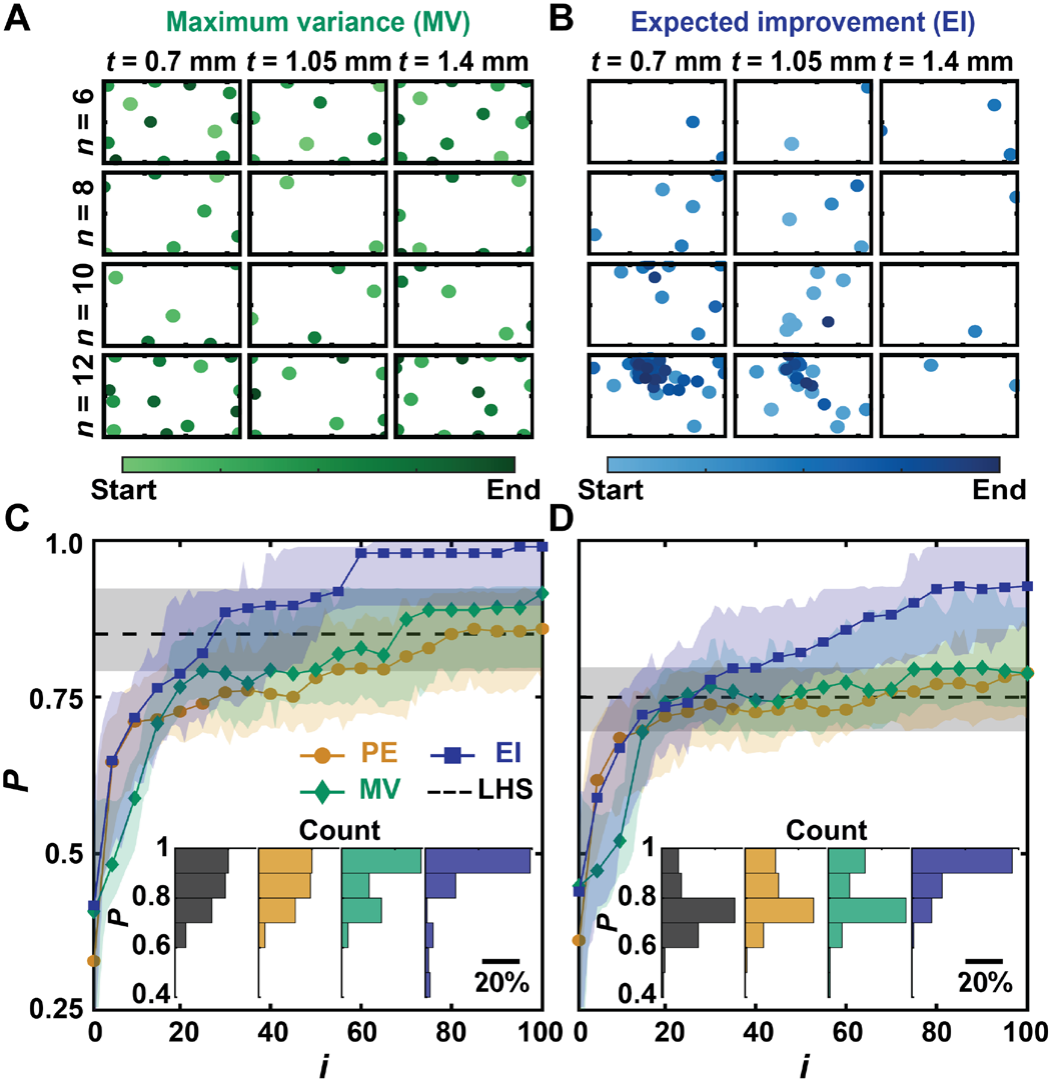
Simulated learning using BO. Distribution of experimental points when guided using (**A**) MV and (**B**) EI decision-making policies. The color gradient indicates the start and end of the campaign. Axis limits are the same as in Fig. 2E. Performance *P* versus experiment number *i* of simulated Bayesian campaigns with noise added to each simulated measurement drawn from a zero-mean Gaussian with (**C**) SD σ = 0.1 J and (**D**) σ = 5 J. EI- and MV-guided campaigns are benchmarked against PE and the average result of selecting 100 experiments using Latin hypercube sampling (LHS). Shaded regions correspond to the middle two quartiles of 100 simulated campaigns. The inset bar charts show the distribution in *P* at *i* = 100.

We studied three principal decision-making policies: pure exploration (PE), maximum variance (MV) (Fig. 3A), and expected improvement (EI) (Fig. 3B). These decision-making policies were selected because of their popularity in the optimization community and their distinctive explorative and exploitative qualities (*25*, *26*). PE is a purely explorative decision-making policy, where each subsequent experiment was chosen randomly. While PE will eventually explore the parameter space and is unlikely to get trapped by local maxima, an appropriate experimental budget is often unknown or too large. The MV decision-making policy also prioritizes exploration but takes the surrogate model into account by choosing experiments in regions with the largest uncertainty. An advantage of this approach is the exploration of undiscovered regions that might have high-performing designs; however, the number of experiments necessary to adequately explore parameter space is also often unknown or too large. The EI decision-making policy is an improvement-based policy in which subsequent experiments are selected on the basis of the likelihood of surpassing previously observed responses. In contrast with MV and PE, EI is more likely to get trapped by local maxima. Purely exploitative policies were not considered because of the use of an uninformative prior in the experimental campaigns.

The performance *P* of a given campaign after *i* experiments was given by its predicted optimum *x_i_* and was defined as *P*(*i*) = *U*_grid_(*x_i_*)/ max (*U*_grid_). On the basis of this definition, *P* = 1 indicated that the campaign had found the optimum design. In the low noise limit (σ = 0.1 J), all policies achieved median performance $\tilde{P}\geq 85\%$ in 100 experiments, with EI achieving $\tilde{P}>98\%$ (Fig. 3C). However, when the noise level was increased (σ = 5 J), EI alone achieved $\tilde{P}>90\%$ within 100 experiments (Fig. 3D). The incomplete convergence in the low noise and high noise limits is a result of the limited experimental budget allotted to the simulated campaigns. As a comparison to BO, we also simulated campaigns based on Latin hypercube sampling (LHS) with the same experimental budget. For the high noise limit, LHS-based campaigns achieved $\tilde{P}=75\%$, which is similar to PE but is inferior to EI-based campaigns. Note that the EI approach used here differs from standard approaches, which have been reported to result in EI being too greedy (*31*), by selecting from a finite number of random candidate experiments to be evaluated by EI. This stochastic approach was seen to markedly improve the convergence of EI-based campaigns (fig. S4).

### Realizing and benchmarking autonomous experimental optimization

**Fig. 4:**
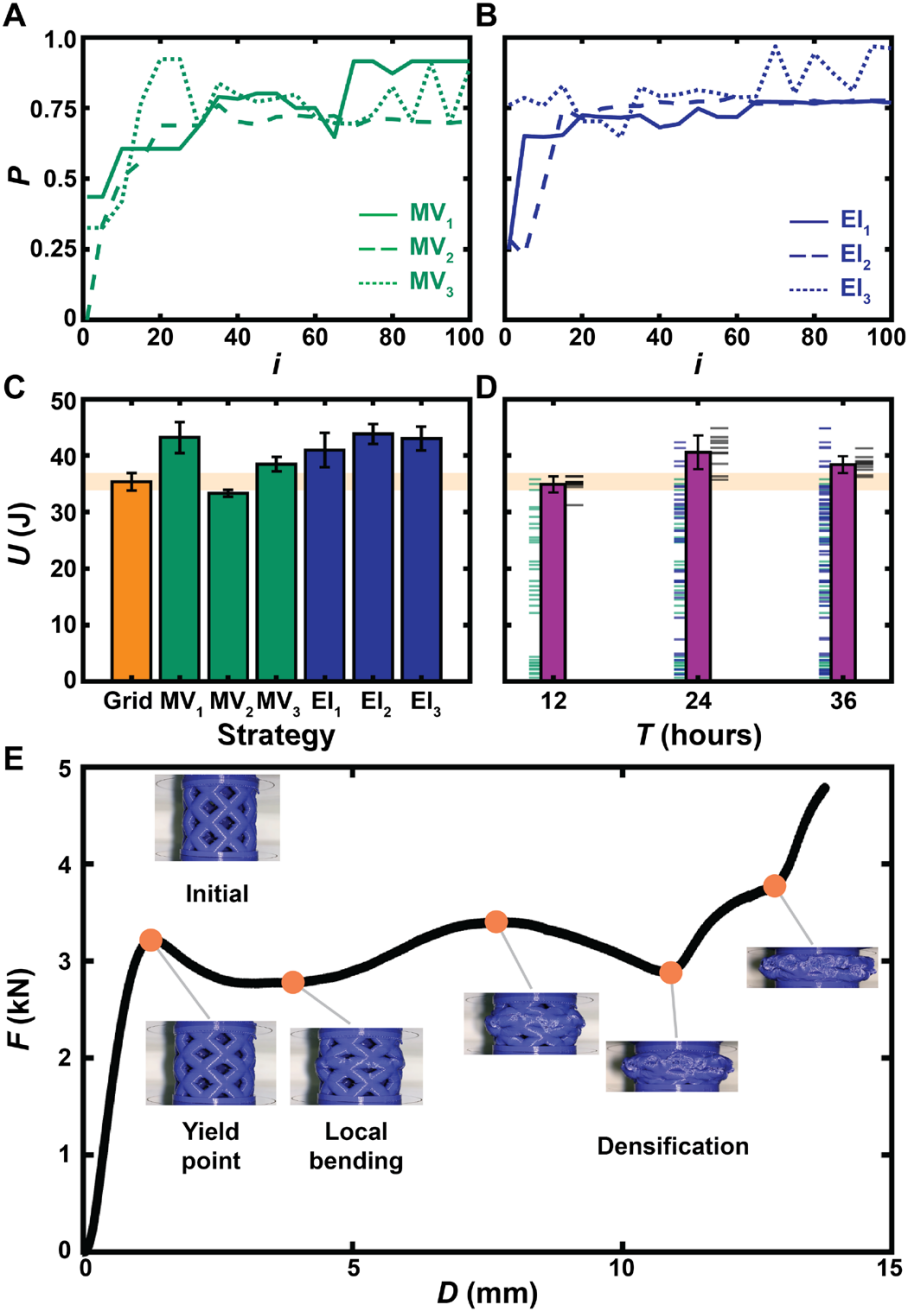
Optimization of a family of mechanical structures using the BEAR. Computed *P* from six experimental campaigns carried out by the BEAR using (**A**) MV and (**B**) EI. (**C**) Average *U* measured from 10 samples of the best predicted structure from each of the six experimental campaigns and the best-performance structure predicted by the grid search. (**D**) Experimental optimization of *U* versus time *T* with ticks to the left of each bar denoting measurements taken before that time, ticks to the right denoting the 10 samples taken at the end of the campaign to evaluate the best predicted sample, and bars denoting the average measurement of the 10 samples. In (C) and (D), error bars correspond to SD. (**E**) Photographs overlaid on the *F* versus *D* curve corresponding to a structure printed with the best-performance design (12, 131°, 1.95 mm, 1.4 mm). (Photo credit: Aldair E. Gongora, Boston University).

While simulation predicts that BO will outperform grid-based approaches, such as LHS, these simulations were based on a number of assumptions, namely, the model of ground truth, the noise profile, and the sampling strategy. Thus, it is imperative to experimentally explore the utility of the BO framework. We therefore integrated the BO framework with the automated research system to produce a BEAR that chose, performed, and learned from experiments. We performed six experimental campaigns, three that were guided by EI and three that were guided by MV. The results of the BEAR’s experimental campaigns were compared with the predicted best-performance structure according to *U*_grid_, where *P*, on average, increased with *i* (Fig. 4, A and B). However, it is worth emphasizing that, first, unlike in the simulated campaigns where *U*_grid_ is treated as ground truth, *U*_grid_ here is a statistically regressed model and, second, the only reliable method to assess the performance of an experimental campaign is to experimentally test the predicted best-performance structure. Here, we accomplished this by testing 10 copies of the optimum structures predicted by each experimental campaign (Fig. 4C). On the basis of the experimental tests, five of the six optimum structures found by the experimental campaigns outperformed the best structure predicted by *U*_grid_, showing that, in five instances—including all three based on the EI decision-making policy—100 well-chosen experiments were superior to 1800 experiments chosen on a grid.

While the BEAR was successful in optimizing the crossed barrel, the superiority of experimental performance (Fig. 4C) over simulated performance (Fig. 4, A and B) was likely because the results of experiment (*U*) differed markedly from the experimentally derived surrogate model (*U*_grid_). Two possible reasons for this discrepancy were insufficient data to build an accurate surrogate model and that the experimental function was observed to be heteroscedastic, while *U*_grid_ was fit using a homoscedastic model. While, in principle, one could perform enough experiments to accurately model the noise profile and obtain a more faithful model of the truth more generally, this would likely require a prohibitive number of experiments. Thus, these results not only highlight the need for experiments in benchmarking optimization strategies but also emphasize the importance of experiment selection when the design space is high dimensional and when building an accurate surrogate model is impossible or impractical. Moreover, these results position uncertainty quantification as an equal partner to balance exploration and exploitation in an optimization process.

While the BEAR was found to be efficient with respect to the number of experiments, it is often more important to be efficient in terms of time when optimizing a property. Thus, we explored how multiple printers could be used in concert to optimize performance in minimal time. We note that using all printers at all times necessitated selecting samples in parallel. The decision-making policy associated with choosing jobs in such a batch system is the topic of current research, with recently developed batch-based EI policies showing promise in simulation (*32*–*35*). The BEAR provides an experimental platform to evaluate these policies. As an initial campaign to serve as a benchmark, we performed a Bayesian experimental campaign in which six agents selected experiments based on the data collected from all agents. While, in theory, EI should balance exploration and exploitation if uncertainties are properly quantified, the use of an initially uninformative prior belief limits uncertainty quantification. In practice, a standard approach is to select a number of randomly chosen samples to explore the parameter space and train the Gaussian process. Here, we spend the first 12 hours of the campaign selecting experiments (32 samples) using MV. MV-guided simulated campaigns show that convergence saturates at ~30 experiments (Fig. 3D), further motivating this practice. After this initial 12-hour period, we switched to an EI decision-making policy. The predicted best-performance structures at *T* = 12, 24, and 36 hours were each experimentally tested to determine their performance. The BEAR matched the toughness from the grid-based experimental campaign (1800 experiments) after 12 hours (32 experiments) and outperformed the grid-based experimental campaign after 24 hours (64 experiments) (Fig. 4D).

Ultimately, the extensive experiments described herein allowed us to identify the member of the crossed barrel family with the largest *U*, namely, (12, 131°, 1.95 mm, 1.4 mm). Inspecting the *F*-*D* curve corresponding to one such sample, a number of interesting features were evident (Fig. 4E). In particular, a series of six inflection points were observed, which corresponded to different mechanical processes including the initial yield point and a series of buckling and reentrant contact modes. Notably, the precise parameter values that produce the largest net positive effect of these sometimes competing and sometimes synergistic processes on *U* would be difficult to predict in the absence of experiment.

## DISCUSSION

The observation that a BEAR can, in the case of optimizing toughness, reduce the number of experiments needed by a factor of almost 60 has potentially far-reaching implications spanning mechanics and the field of autonomous experimentation more broadly. In mechanics, the combination of a BEAR and simulation-based approaches such as topological optimization could allow for the rapid optimization and discovery of novel properties that are difficult or impossible to find using other means. More generally, the use of AM in a BEAR adds a critical degree of versatility that is analogous to the use of automated liquid handling in chemistry. Building an autonomous research system is most justified in fields such as mechanics where even a single AM instrument is versatile enough to allow a wide range of experiments. In this way, a BEAR may have a transformative impact on mechanics. Last, however, it is worth emphasizing that throughout this work, >2500 experiments were spent, proving that only 32 experiments were required to reach an optimal structure. As an emerging field, it is critical that autonomous experimentation provides these benchmarks to illustrate the possible improvement using BEARs. Looking forward, this validation of the transformative acceleration inherent to BEARs, at least in this class of problems, will allow future work to transition from benchmarking to discovery.

Through the combination of a high-throughput automated experimental system and BO to select experiments, we have developed a BEAR that reduced the experimental time and experiments needed to optimize toughness, a mechanical property that is difficult or impossible to simulate. This work is based on (i) a system that combines an array of 3D printers with robotics and testing equipment such that samples can be tested without human intervention and (ii) a BO framework that guides the action of the high-throughput system. In addition to addressing how to effectively choose a decision-making policy when the ground truth function is unknown, the high-throughput nature of this process allowed us to quantify and explore a large parameter space of AM parts. From a learning perspective, realizing a BEAR required advancing several facets including the development of modifications to standard EI algorithms and facile processes for performing BO in batch. Considering the ubiquity of properties that cannot be effectively simulated at present, we anticipate that BEARs based on the principles describe herein could have a transformative impact in mechanics and in fields ranging from chemistry, materials, and biology.

## MATERIALS AND METHODS

All structures were printed using a MakerGear M3 FDM printers out of polylactic acid (PLA). The diameters of the printer nozzle and the PLA filament were 0.35 and 1.75 mm, respectively. The structures were printed with a rectilinear infill pattern at 100% infill. During printing, the printer bed was set to 85°C for the first layer and 75°C for all subsequent layers. The PLA filament was extruded at 215°C. After the print completed, the structures were retrieved when the bed temperature was below 40°C. The structures were uniaxially compressed at a speed of 3 mm/min with a maximum force threshold of 4.8 kN. Toughness was computed as the area under the force-displacement curve, where the force-displacement curve was truncated if the force was below 50 N (1% of the maximum allowable force) after an initial 2 mm of displacement. The threshold was used to avoid including the loads from the compression of fragments of the fractured barrel.

Gaussian process priors in the BO framework were specified with a zero-mean function and a squared exponential covariance kernel, $\sum (x,x')=\alpha^{2}\text{exp}(-\frac{1}{2}\sum_{i=1}^{d}\frac{{\left(x_{i}-{x'}_{i}\right)}^{2}}{\beta_{i}^{2}})$, where *x* = (*n*, θ, *r*, *t*). The kernel is parametrized by *d* + 1 parameters, α, β*_i_*, …, β*_d_*, where *d* = 4 is the dimensionality of the design parameter space. The parameters were initialized as α = 50, β*_i_* = ( max (*x_i_*) − min (*x_i_*))/(10). In addition, the Gaussian process formulation assumed independent, homoscedastic noise, and the SD of the noise was initialized as 5 J. The parameters of the kernel and the noise were optimized using maximum likelihood estimation after every subsequent observation. The parameters in the optimization were bounded to be greater than or less than their individual initial values by a factor of 10 or were individually reset to their initial values. This was performed to avoid obtaining extremal hyperparameters. The decision-making policy selected the next experiment from a uniformly random finite number of candidate designs (fig. S4).

For each autonomous experiment (fig. S1), the crossed barrel design input *x* = (*n*, θ, *r*, *t*) was converted to a standard triangle language (STL) file using OpenSCAD, an open-source software for parametric computer-aided design. The generated STL file was then converted to g-code using Slic3r, an open-source tool for converting a 3D model into g-code. The g-code was then uploaded for 3D printing using OctoPrint, a web-based software to interface with the 3D printer. After the structure was printed, the robotic arm retrieved the structure when the bed temperature was below 40°C. The structure was then weighed on the scale and then tested on the universal testing machine (5965, Instron Inc.). The weight reading, the force-displacement curve, and the computed toughness were all saved to a local database. Using the database, the BEAR built a belief model using GPR and selected the design parameters of the next experiment using a decision-making policy. This process was repeated for a given experiment budget or experiment run time and was operated without a human in the loop. A custom script written in MATLAB was used to coordinate the operation of these components.

## Supplementary Material

aaz1708_SM.pdf

aaz1708_Movie_S1.mp4

## SUPPLEMENTARY MATERIALS

Supplementary material for this article is available at http://advances.sciencemag.org/cgi/content/full/6/15/eaaz1708/DC1
