# Supplementary material for: A Bayesian experimental autonomous researcher for mechanical design
Source: Sci Adv. 2020 Apr 10;6(15):eaaz1708. doi: 10.1126/sciadv.aaz1708 (PMC7148087; doi:10.1126/sciadv.aaz1708)
Supplement: aaz1708_SM.pdf [file aaz1708_SM.pdf]

Science Advances
AAAS

advances.sciencemag.org/cgi/content/full/6/15/eaaz1708/DC1

Supplementary Materials for

A Bayesian experimental autonomous researcher for mechanical design

Aldair E. Gongora, Bowen Xu, Wyatt Perry, Chika Okoye, Patrick Riley, Kristofer G. Reyes*, Elise F. Morgan*, Keith A. Brown*

*Corresponding author. Email: brownka@bu.edu (K.A.B.); efmorgan@bu.edu (E.F.M.); kreyes3@buffalo.edu (K.G.R.)

Published 10 April 2020, *Sci. Adv.* **6**, eaaz1708 (2020)
DOI: 10.1126/sciadv.aaz1708

The PDF file includes:

Figs. S1 to S4
Legend for movie S1

Other Supplementary Material for this manuscript includes the following:

(available at advances.sciencemag.org/cgi/content/full/6/15/eaaz1708/DC1)

Movie S1

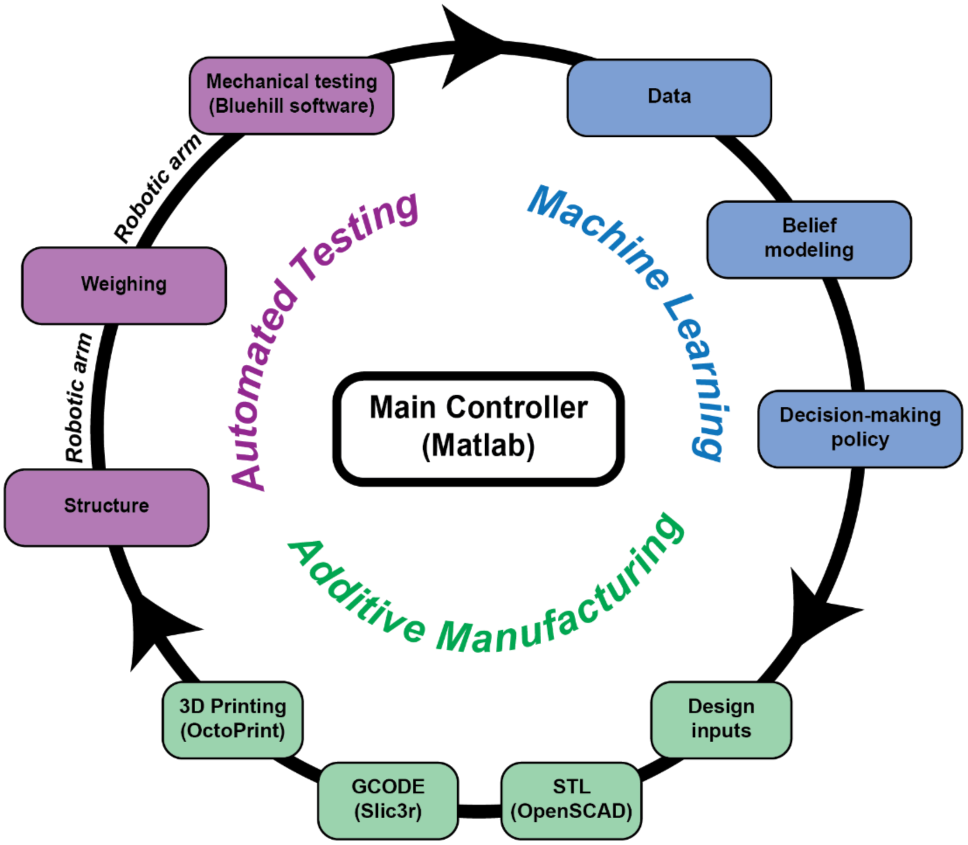

Fig. S1. Framework of the Bayesian experimental autonomous researcher (BEAR).
The autonomous experimental loop includes constructing parts using additive manufacturing, testing them in an automated fashion, and using machine learning to coordinate these actions.

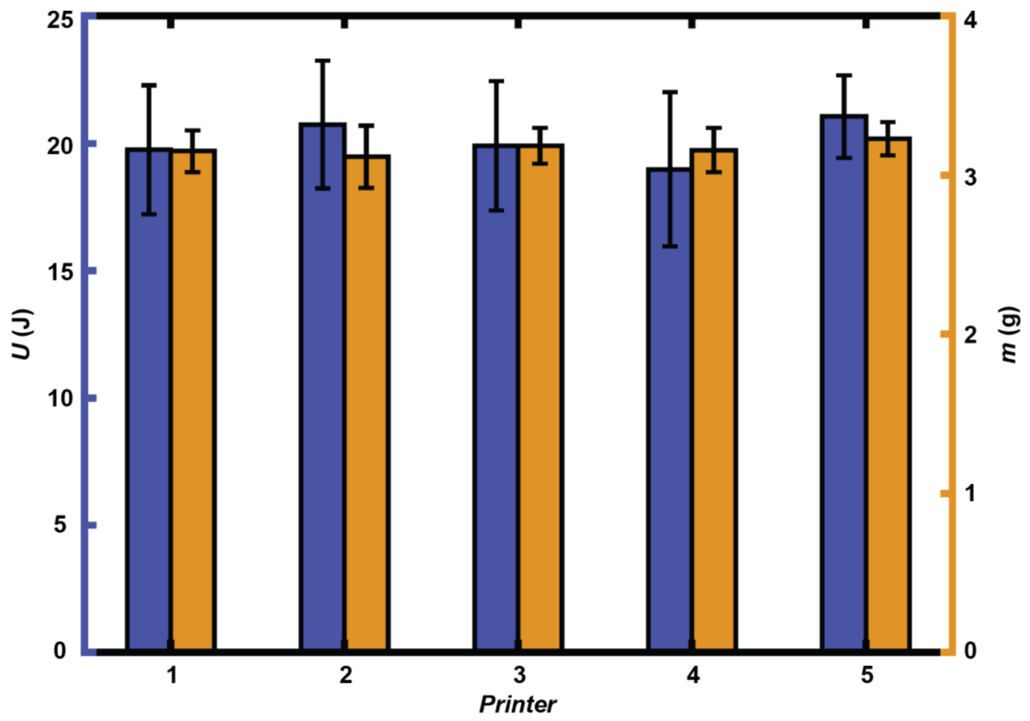

Fig. S2. Printer-specific experimental toughness and mass of samples.

Experimentally observed energy absorption U and mass m from 240 samples written using the same design by five different printers.

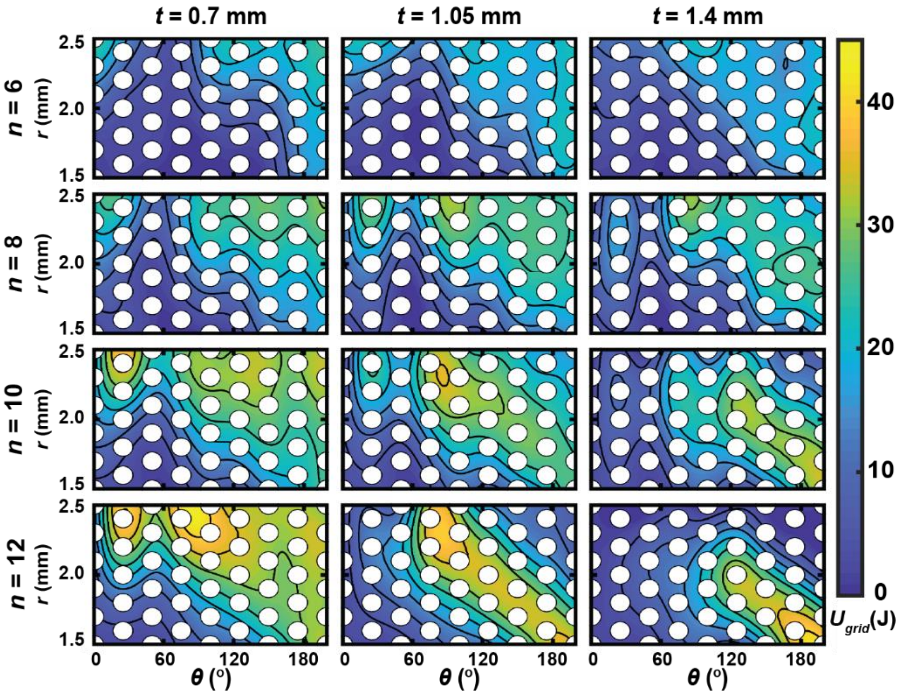

Fig. S3. Surface plot of predicted toughness U_{grid} with grid-search points denoted.

Markers indicate the 600 points sampled by grid-searching. Each point was sampled three times to result in a total of 1,800 samples. The resulting data was used to train a gaussian process U_{grid} which is shown as the color scale.

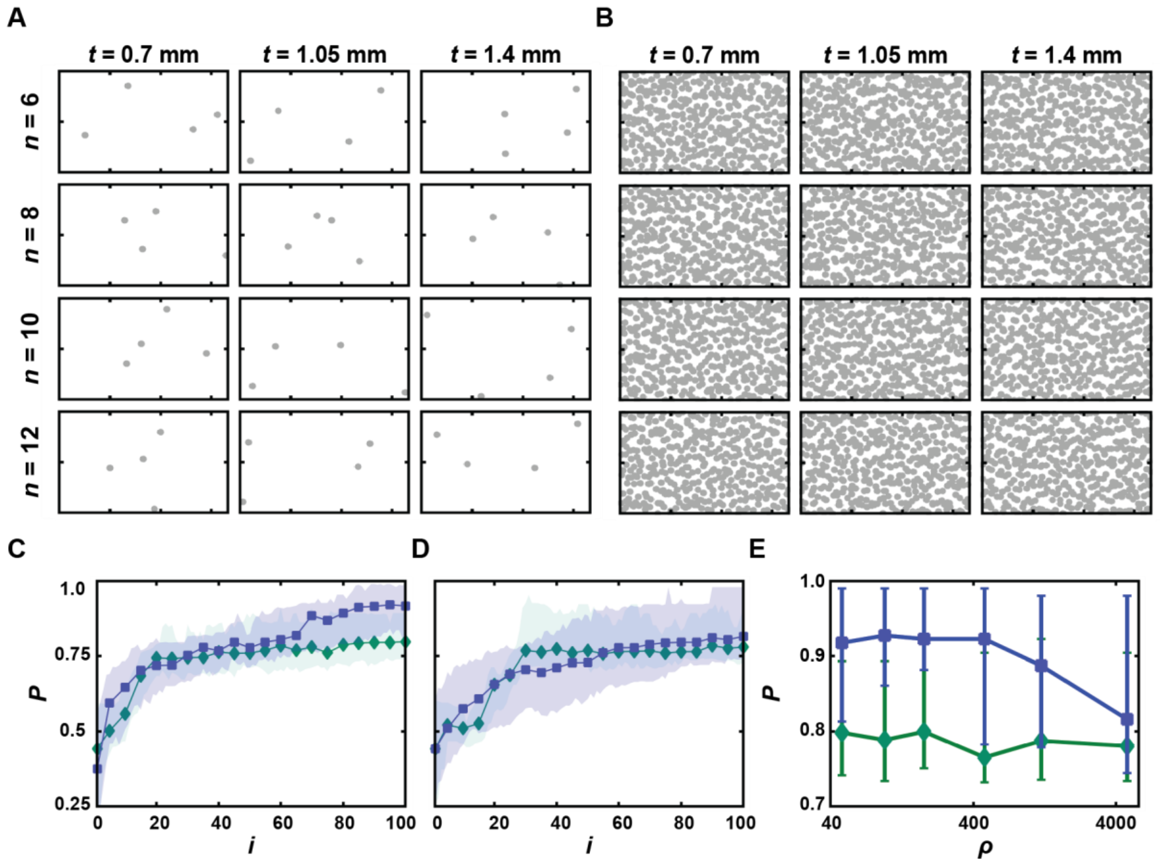

Fig. S4. Simulations of sampling strategies.

Depictions of the designs evaluated when selecting subsequent experiments with (A) a small number (ρ = 48) of candidates and (B) a large number (ρ = 4800) of candidates, where ρ indicates the number of candidate designs. The simulated performance P of maximum variance (MV) and expected improvement with (C) ρ = 48 and (D) ρ = 4800. (E) The performance of MV is not significantly affected by ρ, while performance for EI degrades as ρ increases. Based upon these results, $\rho = 96$ was used for the BEAR.

Movie S1. Automated run of the grid-based search.

An 8-hour video was shot at 0.2 frames per second (fps) and sped up by a factor of 300 to depict the automation capabilities of the system during the grid-based search.
